# Ultra-broad Mid-IR Supercontinuum Generation in Single, Bi and Tri Layer Graphene Nano-Plasmonic waveguides pumping at Low Input Peak Powers

**DOI:** 10.1038/s41598-017-10141-3

**Published:** 2017-08-31

**Authors:** Swetha S. Bobba, Arti Agrawal

**Affiliations:** Department of Electrical and Electronic Engineering, City, University of London, Northampton Square, London, EC1V 0HB UK

## Abstract

This article presents four different plasmonic structures using Graphene which yielded an efficient plasmonic mode with low loss for Supercontinuum(SC) generation. At an operating wavelength of 1550 nm in these structures, we generated a multi-octave broadband SC spectrum ranging from 1.5 um–25 um at a low input peak power of 1 W. Due to pumping in the anomalous dispersion region with two Zero Dispersion Wavelengths (ZDWs) and the process of cross phase modulation with soliton fission, red-shifted dispersive waves were generated which led to large broadening from 1.5 um–25 um. Two other Supercontinua ranging from 1–10 um and 0.85–2.2 um also at low input peak powers of 2 W and 0.1 W respectively were generated. These three supercontinua are useful for applications in the fields of biomedical sensors, spectroscopy, fluorescence lifetime imaging and in the design of many other new optical devices. Furthermore, we have also discussed our results on behaviour of Graphene as a metal, even without the negative real value of dielectric constant.

## Introduction

Supercontinuum Generation (SCG) has been widely studied over the past 40 years. Detailed analysis of non-linear dynamics in solids, organic and inorganic liquids, gases^1^, various types of waveguides and optical fibres^2, 3^ has led to the design of wide-band Supercontinuum light sources. However, they require very high input peak powers ranging from 1 kW to 1000 kW or more^2–9^. From the literature in the area^10–13^, low input peak powers (of the order of Watts) has limited the spectral broadening and thereby their usability for wide range of applications^14^. Dispersion engineered waveguides and/or fibres using different materials have been developed but their main limitation is the control of peak input power with low loss for broadband SCG.

For example, in 2015, Yu *et al*. generated a SC spanning from 1.8 to 10 μm by pumping a Ge-As-Se/Ge-As-S chalcogenide fibre with 330 fs pulses at 4 μm using ~3000 W input^3^. In 2016, a three-layer index guided lead silicate (SF57) photonic crystal fibre^4^ was used to generate three octaves spanning SC from 900 to 7200 nm using 50 fs sech optical pulses of 5 kW peak input power with a large nonlinear coefficient of ~1078 W^−1^ km^−1^. Recently, Cheng *et al*. experimentally demonstrated mid-infrared (MIR) SCG spanning ~2.0 to 15.1 μm in a 3 cm-long chalcogenide step-index fiber with a pulse width of ~170 fs at 9.8 um with input peak power of 2.89 MW^5^. This is the highest average power SC covering 2–15.1 μm reported to date. Also, the broadening beyond 15 um for this design is not possible due to material absorption of the chalcogenide. Our results thus emphasize on solutions to these problems – the peak input power and loss by using Graphene as one of the core materials for the waveguide designs.

Graphene^15^ is a two-dimensional (2D) Carbon material with a honey-comb lattice. Recently Graphene nano-ribbon and other waveguides have emerged showing possible outstanding applications in the fields of on-chip interconnects, bright visible light emission, flexible electronics and more^16, 17^. However, in all these designs, Graphene has been used as the outer core or the cladding material. Thus, from our research, we have shown that with Graphene as the inner core in the designed waveguides, we can exploit its remarkable non-linear properties including a very high Kerr coefficient of –1.1 × 10^−13^ m^2^/W^18^ for broadband SCG at a low input peak power, while exhibiting low material losses.

## Waveguide Design and Implementation

In our design of Graphene waveguides for SCG: the structure of a ridge waveguide rests on a Silicon dioxide (SiO_2_) substrate with the inner and outer core surrounded by a low-index medium(air), thereby providing strong optical confinement.

The inner core of the designed ridge waveguides consists of a single or bi layer of Graphene, of thickness 0.335 nm and 0.67 nm respectively. A material (Si_3_N_4_) with hexagonal crystalline structure capable of controlling the overall waveguide loss, with tunable band-gap properties for electro-optic applications is used as the outer core. This design thereby produces an efficient mode to guide the light leading to broader SC. The low refractive index contrast of the waveguide materials was kept in mind to avoid large non-linear effects which can narrow the Supercontinuum broadening with losses.

Four Graphene waveguide designs (Single, with and without buffer for Bi, and only with Buffer for Tri layers) were simulated using Finite Element Method(FEM)^19, 20^ to calculate the Group Velocity Dispersion (GVD, β_2_) curve and the respective higher order dispersion coefficients (β_3_, β_4_). Supercontinuum for these designs were further calculated by solving the Generalized Non-Linear Schrödinger Equation (GNLSE) using the Split-step Fourier method.

In all the simulations performed, Graphene was tuned to two chemical potentials, µ_c_ = 450 meV and 500 meV, which can be achieved by applying a gate voltage to the 2D material. This range of chemical potentials were specifically used to tune Graphene as a metal in the operating wavelength region (1550 nm) with low waveguide loss, thereby generating a plasmonic mode at the dielectric-metal-dielectric interface.

## Results

**Figure 1:**
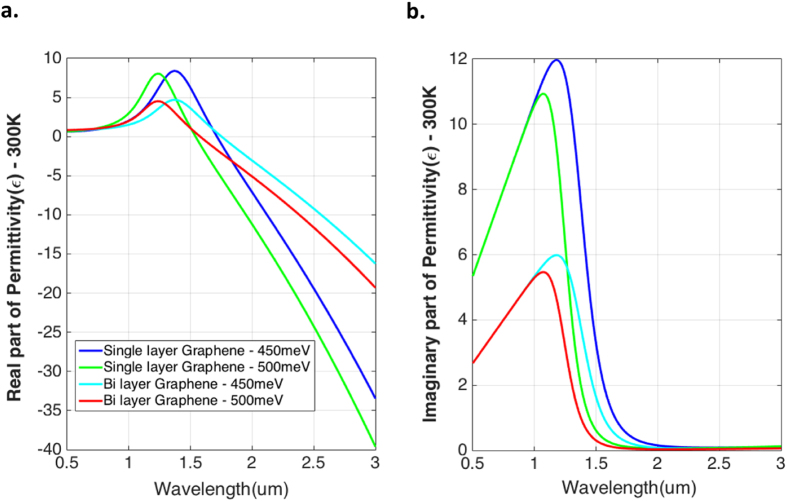
Real and Imaginary part of Permittivity of Graphene plotted from 0.5–3 um. (**a**,**b**), Calculated permittivity of Graphene at two different chemical potentials and temperatures of 450 meV and 500 meV at 300 K. These results are benchmarked with^50^.

The Conductivity (σ_g_) and Permittivity (ɛ_g_) of single and bi layer of Graphene were calculated from the Kubo formula^21–23^ using a FORTRAN code developed by us. The wavelength variation of the real and imaginary parts of the permittivity is shown in Fig. 1a and b, at 450 and 500 meV, 300 K. Table 1 shows these values for single layer of Graphene operating at 1550 nm wavelength (at 300 K and 371 K).

**Table 1:** Calculated conductivity and permittivity of single layer Graphene at 1550 nm for 450 meV and 500 meV - 300 K and 371 K.

Temperature (T)	Chemical Potential (µ_c_)	Operating Wavelength (λ)	σ_g_ (Conductivity of Graphene)	ɛ_g_ (Permittivity)
300 K	450 meV	1.55 um	7.78289151434 × 10^−06^–1.322691198868 × 10^−05^ S	4.6233 + 2.1320i
371 K	450 meV	1.55 um	1.05877013296 × 10^−05^–1.231768904974 × 10^−05^ S	4.3742 + 2.9003i
300 K	500 meV	1.55 um	1.3906881552 × 10^−06^ + 4.9808553475 × 10^−06^ S	−0.3644 + 0.3810i
371 K	500 meV	1.55 um	2.685510752162 × 10^−06^ + 4.204475290258 × 10^−06^S	−0.1517 + 0.7356i

**Figure 2:**
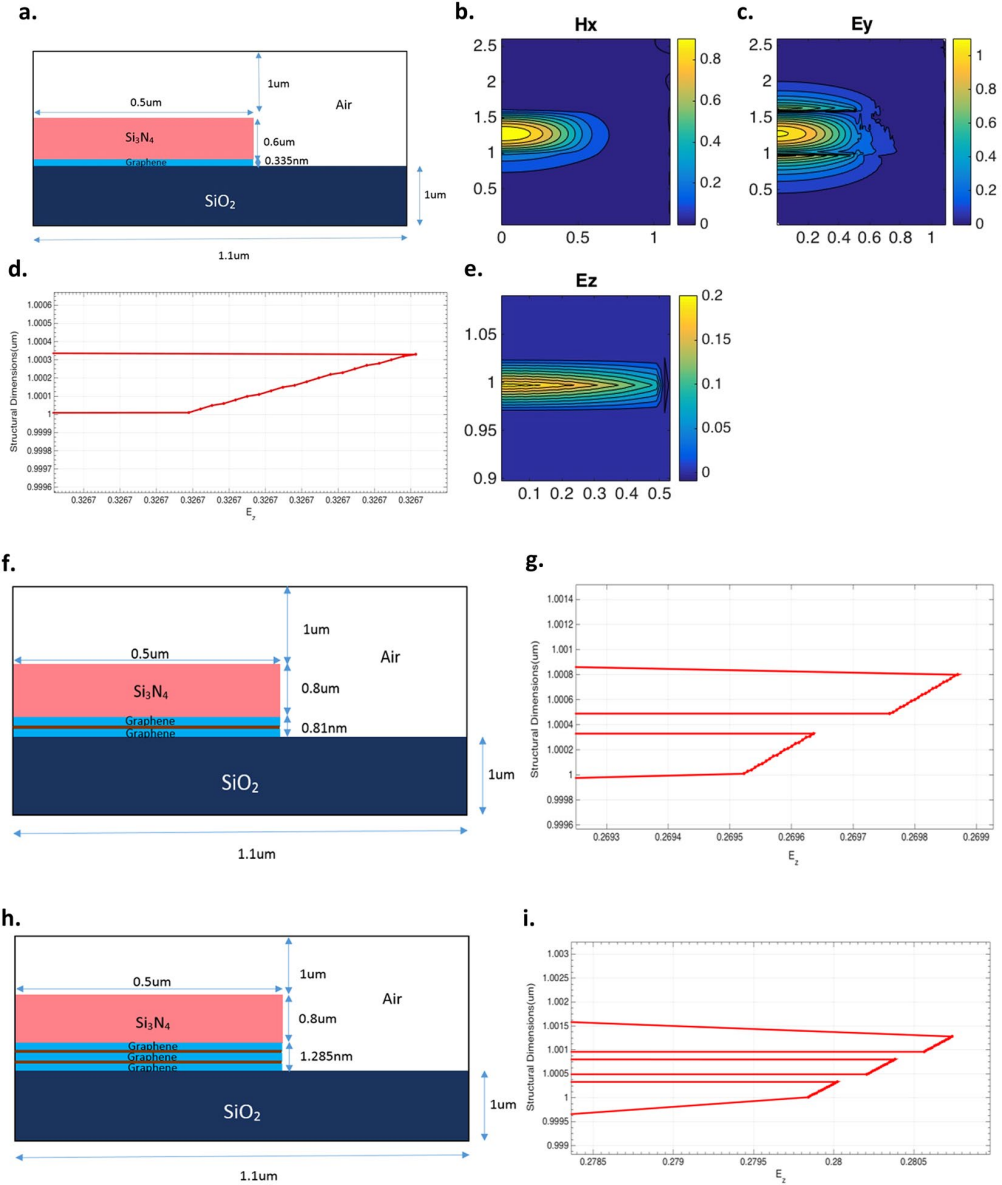
Plasmonic modes generated in the Graphene layer of the designed waveguides at 450 meV and 300 K. (**a**–**e**), Structure of the single layer Graphene Waveguide with dominant field components H_x_, E_y_, E_z_ and, plasmonic mode (line plot of E_z_) on the left. (**f**,**h**), Bi-layer and Tri-layer Graphene waveguides sandwiched with Al_2_O_3_ buffer layer (region shaded brown) of thickness 0.14 nm. (**g**,**i**) The plasmonic modes of bi and tri layer Graphene waveguides on the right.

The waveguide with a single layer of Graphene (﻿Fig. 2a) with complex permittivity calculated at 450 meV, 300 K (﻿from Table 1) was used in the half-waveguide cross section of 1.1 µm wide × 2.600335 µm height to guide the fundamental TM mode initially. The outer core - Si_3_N_4_(Silicon Nitride) is used with permittivity, *ε*_*Si*3N4_ = 6.0945364 on top of the Graphene layer with Silica (SiO_2_), *ε*_*SiO2*_ = 2.0852 as the substrate, and Air, *ε*_*Air*_ = 1 for the cladding operating at 1550 nm. Figure 2b,c and e shows the dominant H_x_, E_y_ and E_z_ field components generated in this structure with the plasmonic mode shown in Fig. 2d. Numerical simulations were then extended to bi (with buffer) and tri (with buffer) layer waveguides as shown in Fig. 2f and h at a chemical potential of 450 meV and temperature of 300 K. It is apparent from the field representation in E_z_ and the plasmonic mode plots from Fig. 2d,g and i, that the mode is highly confined in the inner core of the designed Graphene ridge waveguides and is a plasmonic mode.

The plasmonic mode^24, 25^ in Graphene layer of the designed waveguide geometries is formed from coupling between Surface Plasmon Polaritons (SPPs) generated on the top and bottom interfaces of the 2D material with Silicon Nitride and Silica layers respectively. Thickness of Graphene^22, 26^ also plays a vital role in tight electric field localization at the metal interface, thereby guiding the EM energy with sub-wavelength confinement to micron/sub-micron propagation lengths. Similar physics but with slightly different values of GVD, A_eff_ and loss was observed in these waveguide designs at 450 meV, 371 K, with plasmonic mode-shifting from z to y - axis at 500 meV–300 K and 371 K. This property makes these designs sensitive for sensor applications^26^ that will be investigated in the future.

**Figure 3:**
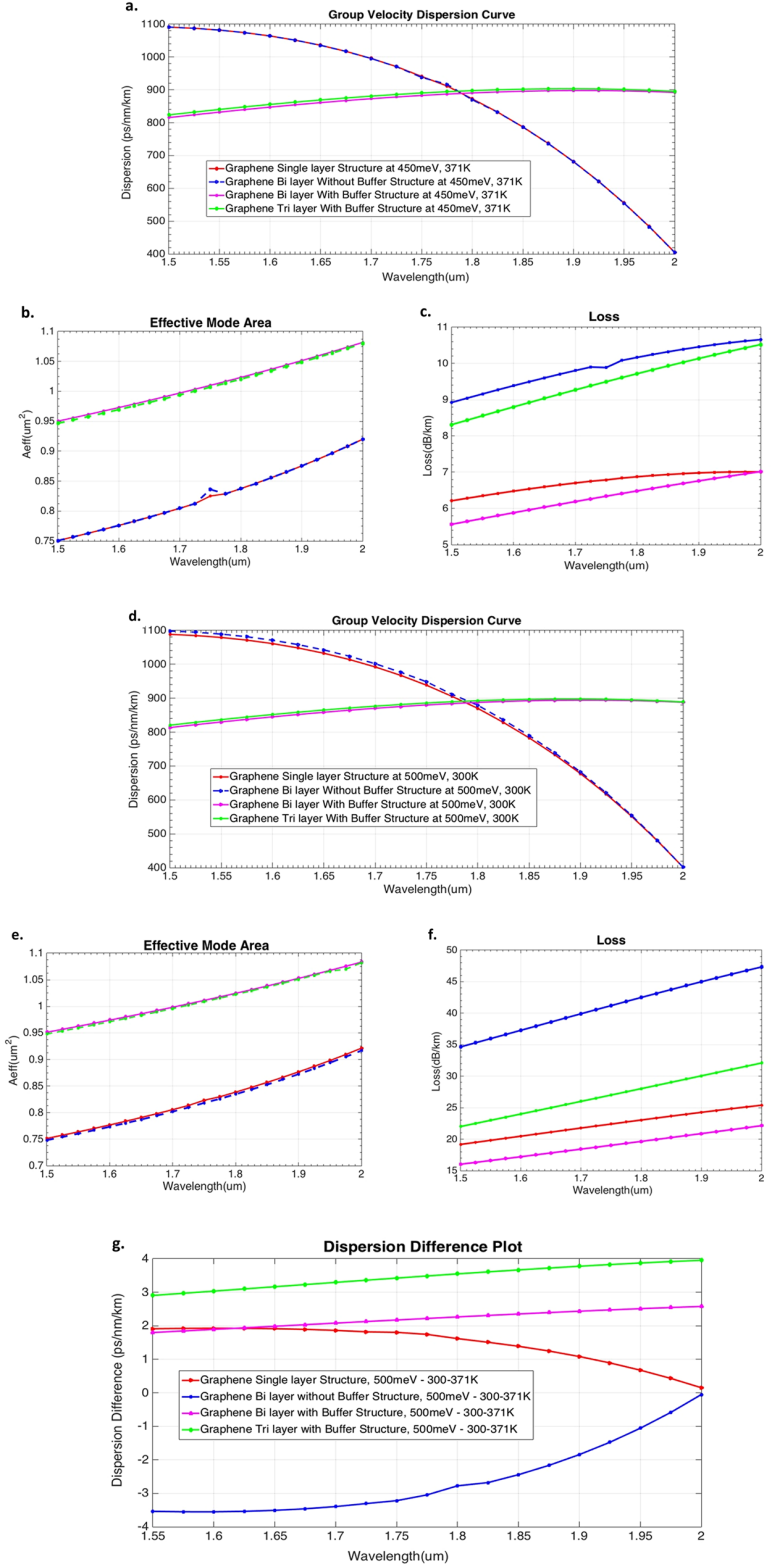
GVD, Effective mode Area and Loss plots for all the designed Graphene waveguides. (**a**–**c**) Calculated GVD curve, A_eff_ and loss of four different Graphene waveguides at 450 meV, 371 K. (**d**–**f**) Calculated GVD curve, A_eff_ and loss of four different Graphene waveguides at 500 meV, 300 K. (**g**) Difference between calculated GVDs at 500 meV, 300 K & 371 K.

Figure 3a–f show the GVD curve (β_2_), effective mode area (A_eff_) and loss plots calculated at a step size of 0.025 µm from 1.5–2 µm wavelength range for all the four designed waveguide structures at 450 meV, 371 K and 500 meV, 300 K respectively. The GVD Curves^27^ show the presence of increasing and decreasing slopes after reaching a peak dispersion value which results in two ZDWs.

At 1.55 um, bi and tri layer graphene waveguides induced a total effective area of 0.962449 µm^2^ and 0.9593657 µm^2^ at 300 K, and 0.9613992 µm^2^ and 0.9578994 µm^2^ at 371 K, for 500 meV chemical potential. These structures also showed an average loss of only ~15 dB/km. The overall dispersion in bi and tri layer structures (with buffer) was moderate with excellent A_eff_ and loss, thereby making them highly [Comment: ﻿The below sentence is﻿ continuation from the ﻿above paragraph, as in, the sentence should read “thereby making them highly favourable for SCG”]favourable for SCG. Similar properties of this GVD curve in Fig. 3a and d was observed at 450 meV, 300 K and 500 meV, 371 K respectively, but with a very small difference of GVD as plotted in Fig. 3g.

### SC Generation

**Figure 4:**
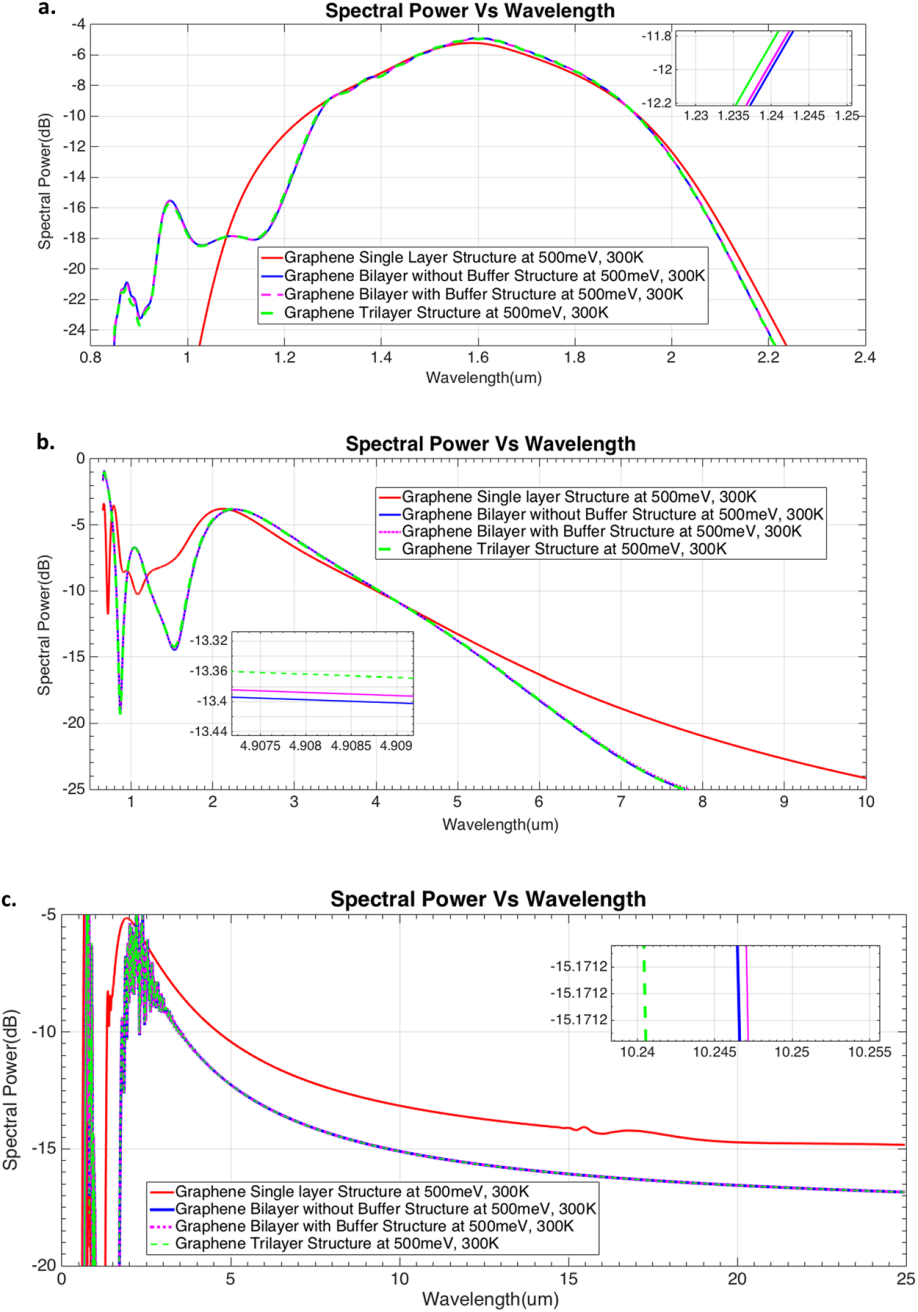
Supercontinuum generation in the designed Graphene waveguides. (**a**–**c**) Spectral Power plotted vs Wavelength for four designed Graphene waveguides at different peak input powers of 0.1W, 2W and 1W respectively (at a chemical potential of 500 meV, 300 K). Three different SC’s were generated at these three input Powers, with varying parameters of pulse width and waveguide length. Zoomed plots in inset show the difference in Supercontinuum for each of the different structures at a given input power.

Supercontinuum in the designed Graphene waveguides was generated operating at 1550 nm, at a pulse width of 10, 2.5 and 1fs for 1 mm, 10 um and 1 mm long waveguides as shown in Fig. 4a,b and c respectively. This generated three Supercontinua, one with multi-octave broadband spectra ranging from 1.5 um–25 um (﻿Fig. 4c) at a very low input peak power of 1 W, thereby making it the broadest Supercontinuum to the best of our knowledge at low input power of the order of 1 W. Two other Supercontinua (﻿Fig. 4b and c) ranging from 1–10 um and 0.85–2.2 um also at very low input peak powers of 2 W and 0.1 W respectively were generated using these designs. Figure 4a–c show that the SC generated (at low input peak powers) in the designed Graphene waveguides exhibit flat spectral broadening (upto 20 dB).

## Discussion

The broad expansion of the SC in Fig. 4c to such a large bandwidth in our design is mainly due to the large non-linear Kerr coefficient of Graphene of the order of ~10^−13^ m^2^/W. This Kerr coefficient parameter^28^ combined with the tailored dispersion of the waveguide generated fundamental optical solitons thereby stimulating dispersive wave radiation in the anomalous dispersion region. The spectral expansion is mainly due to those successively ejected fundamental solitons from the input pulse^29, 30^ during soliton fission.

The largest spectral bandwidth is observed in the Graphene Single layer structure shown in Fig. 4c. However, considering the losses calculated for the four designed waveguides shown in Fig. 3c,f, we find that the Graphene Bi layer with Buffer (at 500 meV, 300 K) structure is also an efficient waveguide design for SCG. We present further results on the dynamics of this SC^31, 32^ in the following material.

**Figure 5:**
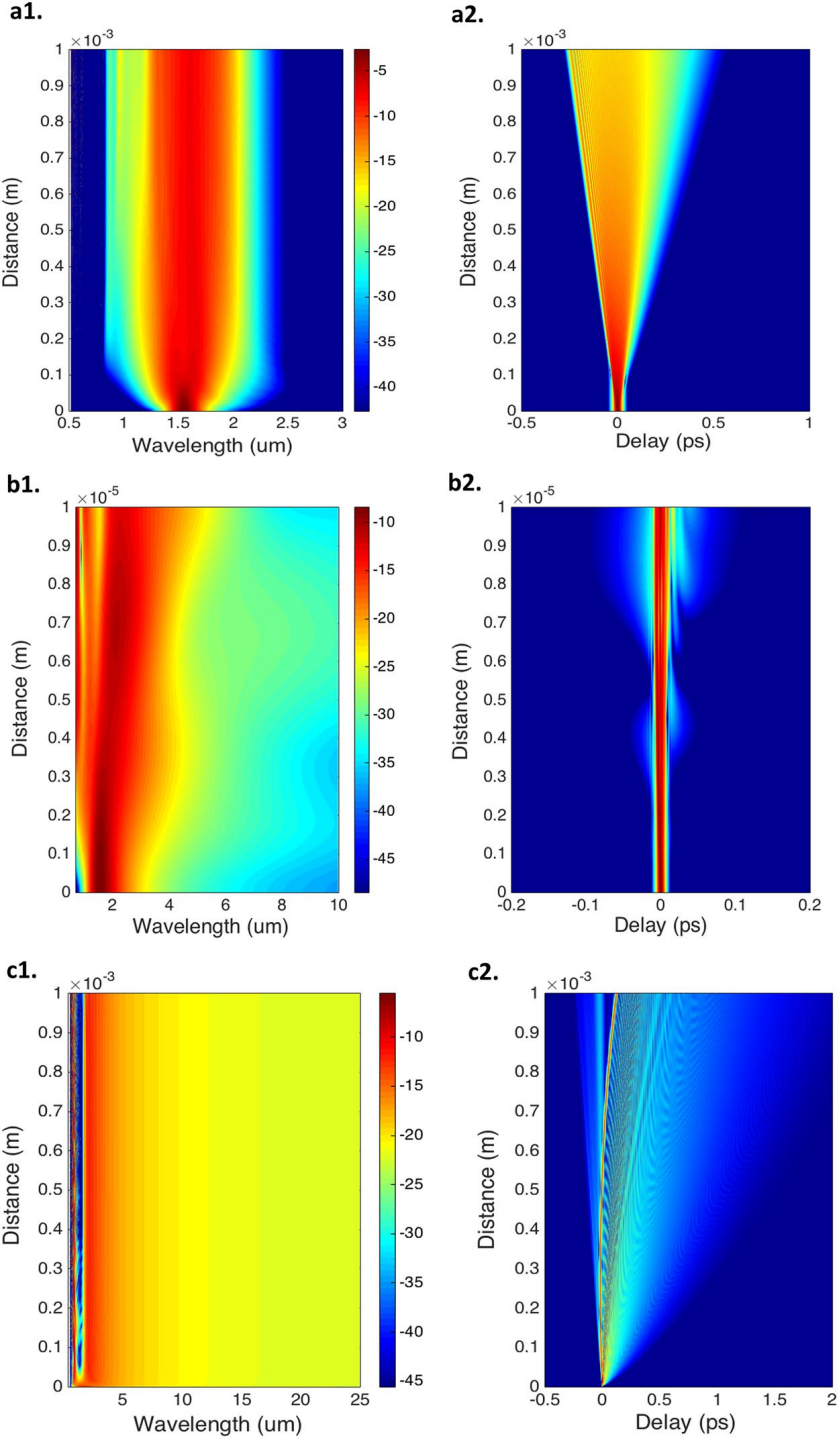
Calculated Spectral and Temporal evolution for the Graphene bi layer with buffer waveguide. **a1**–**c1**, Spectral and **a2–c2**, Temporal evolution of Supercontinuum for the designed Graphene bilayer with buffer waveguide (at 500meV, 300K) with pulse width of 10fs, 2.5fs and 1fs at three input peak powers of 0.1W, 2W and 1W respectively.

For the 1 mm-long Graphene waveguide at a pulse width of 10fs, shown in Fig. 5a1, the nonlinear length is calculated using^31^ L_NL_ = 1/(γP_0_), where γ is the nonlinear coefficient and P_0_ is the peak input power. From calculations, we get L_NL_ at the 1550 nm pump wavelength as 21.584 μm. The dispersion length is L_D_ = T_0_^2^/|β_2_|, where T_0_ is the pulse width and β_2_ = −1.068446552230430 ps^2^∕m at 1.55 μm is the dispersion parameter calculated from Fig. 3. For the peak input power of 0.1 W, L_NL_ is 21.584 × 10^−6^ m, and L_D_ is 9.3594 × 10^−5^ m. Also, the characteristic propagation distance (≈5L_D_) where the ejected soliton separation begins to become apparent in the temporal and spectral characteristic is calculated as ≈ 4.6797 × 10^−4^m. Since the waveguide length L = 1 mm is slightly larger than 5L_D_ and is greater than both L_NL_ and L_D_, the spectrum leads to a number of ejected solitons appearing with a significant decrease of spectral energy in the vicinity of the pump thereby limiting the spectral bandwidth^30^. The soliton order(N) of the waveguide for these input parameters is calculated as ~2.0824.

From Fig. 5b1, for the 10 um long waveguide at a pulse width of 2.5fs, L_NL_ and L_D_ are calculated as 1.0792 um and 5.8496 um respectively with the characteristic propagation distance (≈5L_D_) as 29.248 um. In this case, the waveguide length L = 10 um is much smaller compared to 5L_D_ resulting in only one clearly separated soliton peak in the temporal intensity despite the calculated soliton order(N) being ~2.3281.

For the broadest Supercontinuum generated^31^ from Fig. 5c1 for the 1 mm long waveguide at a pulse width of 1fs, the L_NL_ and L_D_ are calculated as 2.1584 um and 0.93594 um with the characteristic propagation distance calculated as ≈5L_D_ = 4.6797 um. The waveguide length (L) of 1 mm for this design is much larger compared to the 5L_D_ resulting in a greater number of ejected solitons with distinct spectral peaks in the anomalous GVD regime that can be clearly identified as due to soliton fission^33^. The extension of the spectral broadening to the long wavelengths is however due to the generation of red shifted waves formed by coupling of negative dispersion slope (in the vicinity of the second ZDW, the D slope is negative) with the anomalous GVD.

As the pulse width is less than 12fs for the generated SCs above, dispersive perturbation induces soliton fission and this can be modified in two ways. Primarily, as an ejected fundamental soliton shifts to longer wavelengths because of the Raman effect which is small in our case, the soliton encounters a varying value of β_2_, and its temporal width and peak power adjust themselves to conserve a unit soliton number during propagation. This effect is clearly seen in the broader Supercontinuum generated in Fig. 5c1,c2. The flatness of the spectrum is however achieved due to the low third order dispersion (β_3_ = 2.251048248652541 × 10^−3^ ps^3^/m) for this design at 1550 nm.

The second effect is due to the presence of higher-order dispersion which also leads to the transfer of energy from soliton to a narrow-band resonance in the normal GVD regime^13^. This is clearly seen in Fig. 5a1,b1 although the effect is quite small in SC generated in Fig. 5b1 (Fig. 5a2,b2 show their respective temporal evolution of SC). However, this energy transfer decreases over the first few millimetres of propagation thereby limiting the non-linear spectral broadening^34–36^.

These generated Supercontinua have many practical applications such as in DNA sensing, speed/pressure sensor monitoring and medical therapeutics as they cover the full functional band of lipids, proteins, genes, bacteria, viruses, chemicals and spectral region of most biomolecules^37, 38^. The selected Graphene bilayer with buffer structure can be tuned^39, 40^ to 500 meV and practically fabricated by exfoliating graphene and defining its contacts around a particular flake using standard photolithography methods^41–43^. An alternative method for this fabrication is to define the contacts first, transfer large area CVD Graphene on top, then add another layer of photoresist and use O_2_ plasma to isolate devices^44, 45^.

From our results, an important observation is the behaviour of Graphene as a metal, and formation of SPPs on interaction with a dielectric at optical frequencies even without the negative real permittivity value of Graphene at 450 meV (300 K and 371 K temperature). This is different from the theory on Surface Plasmons^46, 47^ which tells us that at a metal-dielectric interface, SPPs are formed when $\frac{{k}_{2}}{{k}_{1}}=-\frac{{\varepsilon }_{2}}{{\varepsilon }_{1}}$, where *ε*_1_ and ${\varepsilon }_{2}$ are the permittivity of the metal and dielectric respectively. In our case, the permittivity of Graphene is not negative, however we still observe the formation of SPPs. This needs further analysis to confirm the behaviour of Graphene and could possibly open doors to a new understanding of Graphene and metals for new applications.

## Conclusion

We have designed four waveguide structures using Graphene as the inner core, with Si_3_N_4_ of thickness - 0.6 um and 0.8 um for the outer core. This fundamentally tailored the plasmonic mode in the dielectric-metal-dielectric interface. With typical waveguide parameters, we achieved a large negative non-linear coefficient of γ = −4.6330088494 × 10^5^ 1/m-W due to negative non-linear Kerr coefficient of Graphene of the order, n_2_ = −1.1 × 10^−13^ m^2^/W that played a major role in stimulating a small Raman effect and thereby leading to broader Supercontinuum by the influence of solitons and dispersive waves.

We have shown that the Graphene Bilayer with Buffer waveguide (at 500 meV, 300 K) exhibits a SC extending from 1.5–25 um at a very low input peak power of 1 W, with overall waveguide loss of only 16.5 dB/km. This is the broadest SC to the best of our knowledge.

## Methods

The modal solutions in the designed Graphene waveguides were obtained using Full Vectorial H-field formulation with penalty term to eliminate the spurious solution. It is one of the most accurate and numerically efficient approaches to obtain the modal field profiles of a waveguide. Various quasi-TE and quasi-TM modes was calculated from equation (1),

1 $${\omega }^{2}=\frac{\iint [{(\nabla \times H)}^{\ast }.{\mathop{\varepsilon }\limits^{\frown {}}}^{-1}(\nabla \times H)+(\frac{\alpha }{\varepsilon })(\nabla .H)\ast (\nabla .H)]\,d\Omega }{\iint {H}^{\ast }.\mathop{\mu }\limits^{\frown {}}Hd\Omega }$$

from which the mode propagation constant β(ω) of the fundamental mode over a range of wavelengths was evaluated, and the effective index was calculated using equation (2),

2 $${N}_{eff}=\frac{\beta (\omega )}{2\pi }\lambda $$

As chromatic dispersion of the waveguide manifests through the wavelength dependence of the refractive index n(λ) (﻿approximated by the refractive index equation, $n=\frac{c}{v}$, where c is the velocity of light in vacuum and v is the velocity of light in medium), the GVD parameter of the Graphene waveguide was calculated from the N_eff_ by equation (3),

3 $$D(\lambda )=\frac{d{\beta }_{1}}{d\omega }=-\frac{\lambda }{c}\frac{{d}^{2}{n}_{eff}}{d{\lambda }^{2}}=-\frac{2\pi c}{{\lambda }^{2}}{\beta }_{2}(\frac{ps}{\frac{nm}{km}})$$

Where ${\beta }_{1}=\frac{1}{{v}_{g}},{v}_{g}$ is the group velocity of pulse envelop and the third-order dispersion (β_3_) was calculated using equation (4),

4 $${\beta }_{3}=\frac{dD(\lambda )}{d\lambda }(\frac{p{s}^{3}}{m})$$

The GVD (Group velocity Dispersion), TOD (Third order Dispersion) and subsequently other higher-order dispersion coefficients were calculated from N_eff_. This calculation was written as a MATLAB code and benchmarked with the material dispersion of Silicon to test its accuracy.

For SCG, the Generalized Non-Linear Schrödinger Equation (GNLSE) was solved using the Split-step Fourier method using equation (5),

5 $$\frac{\partial A}{\partial z}+\frac{\alpha }{2}A-\sum _{k\ge 2}\frac{{i}^{k+1}}{k!}{\beta }_{k}\frac{{\partial }^{k}A}{\partial {T}^{k}}=i\gamma (1+i{\tau }_{shock}\frac{\partial }{\partial T})(A(z,t){{\int }_{-\infty }^{+\infty }R({T}^{\iota })\times |A(z,T-{T}^{\iota })|}^{2}d{T}^{\iota }+i{\gamma }_{R}(z,T))$$

The left side of the equation (5) models the linear propagation effects while the right side models the non-linear effects. This code was incorporated from Dudley *et al*.^48^, moderated and tested for accuracy and efficiency with experimental studies. The non-linear coefficient (γ) in equation (5) is calculated using equation (6),

6 $$\gamma =\frac{2\pi {n}_{2}}{{\lambda }_{o}{A}_{eff}}$$

where n_2_ is the non-linear Kerr coefficient, 𝜆_0_ is the pump wavelength and A_eff_ is the effective mode area.

The results of FEM have been benchmarked^49^ as well with the experimental analysis of other metals to confirm the behaviour of Graphene as a metal at 450 meV (300 K and 371 K).
