# Author Correction: Ultra-broad Mid-IR Supercontinuum Generation in Single, Bi and Tri Layer Graphene Nano-Plasmonic waveguides pumping at Low Input Peak Powers

**DOI:** 10.1038/s41598-019-39692-3

**Published:** 2019-04-10

**Authors:** Swetha S. Bobba, Arti Agrawal

**Affiliations:** Department of Electrical and Electronic Engineering, City, University of London, Northampton Square, London, EC1V 0HB UK

Correction to: *Scientific Reports* 10.1038/s41598-017-10141-3, published online 31 August 2017

In the original version of this Article, Arti Agrawal was listed as corresponding author. Both authors now agree that Swetha S. Bobba should be considered the corresponding author. The PDF and HTML versions of the article have been modified to reflect this change.

